# Supplementary material for: Evolutionary echoes of emotion: Humans mimic other primate expressions
Source: PLoS One. 2026 Mar 11;21(3):e0342196. doi: 10.1371/journal.pone.0342196 (PMC12978439; doi:10.1371/journal.pone.0342196)
Supplement: S1 File — This is the markdown for the R analyses. (PDF) [file pone.0342196.s001.pdf]

R code, results and figures for analyses presented in the main text

Ursula Hess

2026-01-02

Contents

1 Sociodemographics **2**

2 Dimensional emotion ratings **3**
2.1 Graph 3
2.2 Summary statistics for dimensional ratings 3
2.3 Analyses 4

3 Emotion profile **5**
3.1 Summary statistics 5
3.2 Graph 5
3.3 Analysis and post-hocs 7

4 Participant expression (action units) **9**
4.1 Graph 9
4.2 Analysis 10
4.3 Post hoc Helmert contrasts 10

5 Liking and IOS **11**
5.1 Graph 11
5.2 Summary statistics 11
5.3 Analyses 12

6 Predicting the Positive Pattern Score from dimensional emotion ratings **12**
6.1 Summary statistics Positive Pattern Score 12
6.2 Graph 12

7 Using IOS as second predictor **14**

7.1 Analysis 14

7.2 Graph model 14

7.3 Post-hoc analysis 15

8 Powercurve **16**

1 Sociodemographics

Table 1: Age

Statistic	Value
Mean	40.19
SD	13.10

Table 2: Gender Distribution

Gender	Frequency	Percentage
Women	103	49
Men	107	50
Other	0	0
Unknown	2	1

2 Dimensional emotion ratings

2.1 Graph

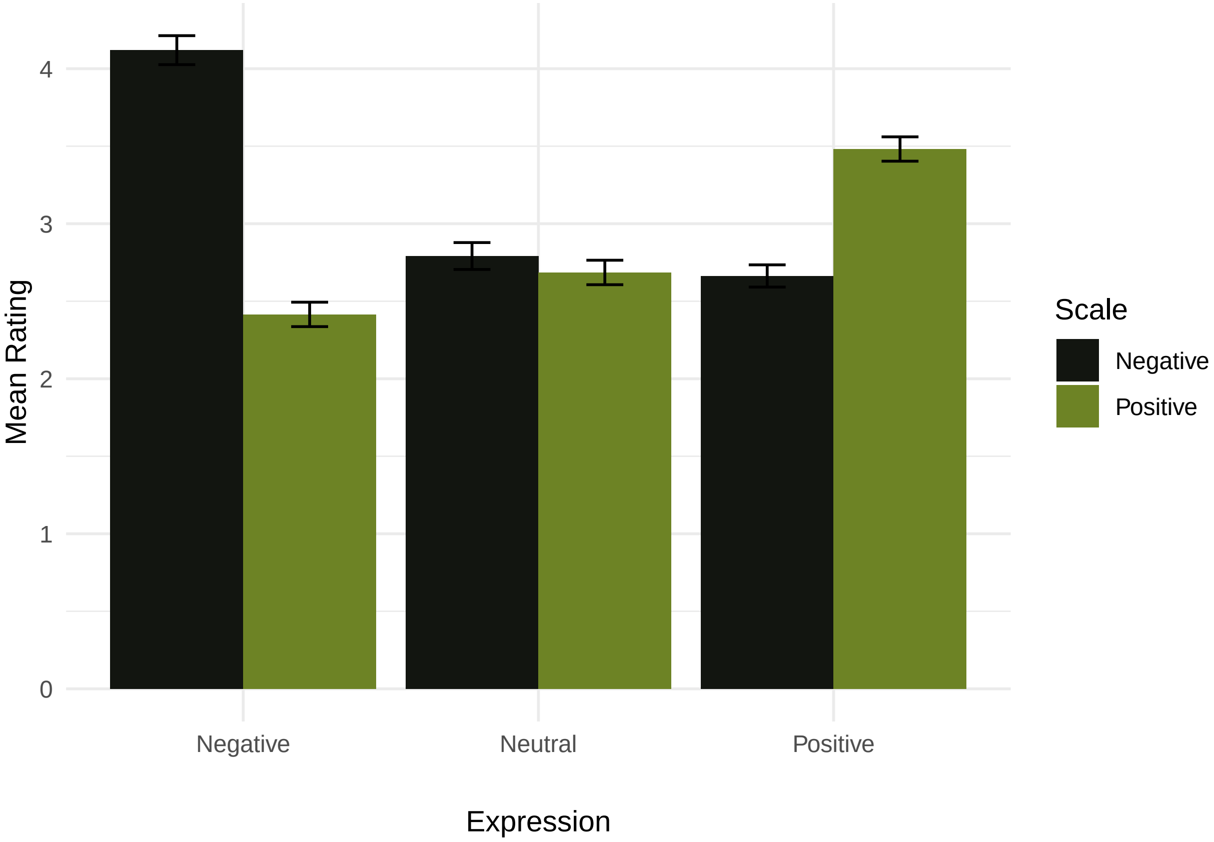

2.2 Summary statistics for dimensional ratings

Table 3: Descriptive statistics by Scale and Expression

Scale	Expression	Mean	SD	CI95 lower	CI95 upper
**Negative**	Negative	4.12	1.31	3.94	4.30
**Negative**	Neutral	2.79	1.23	2.62	2.96
**Negative**	Positive	2.66	1.02	2.52	2.80
**Positive**	Negative	2.42	1.10	2.26	2.57
**Positive**	Neutral	2.69	1.12	2.53	2.84
**Positive**	Positive	3.48	1.11	3.33	3.64

2.3 Analyses

2.3.1 Positivity Ratings

Table 4: ANOVA Results with Partial Eta Squared

	Sum Sq	Mean Sq	NumDF	DenDF	F value	Pr(>F)	Partial ^{2}
Expression	477.3282	238.6641	2	1976.76	81.4403	0	0.0761

Table 5: Pairwise Comparisons

Contrast	Estimate	SE	df	t-ratio	p-value
Negative - Neutral	-0.240	0.091	1978.455	-2.627	0.024
Negative - Positive	-1.103	0.091	1977.493	-12.109	0.000
Neutral - Positive	-0.863	0.091	1977.184	-9.493	0.000

2.3.2 Negativity Ratings

Table 6: ANOVA Results with Partial Eta Squared

	Sum Sq	Mean Sq	NumDF	DenDF	F value	Pr(>F)	Partial ^{2}
Expression	904.389	452.1945	2	1978.795	148.0053	0	0.1301

Table 7: Pairwise Comparisons

Contrast	Estimate	SE	df	t-ratio	p-value
Negative - Neutral	1.314	0.093	1977.339	14.063	0.000
Negative - Positive	1.456	0.093	1976.513	15.651	0.000
Neutral - Positive	0.142	0.093	1976.449	1.534	0.275

Table 8: Descriptive statistics by Emotion Scale and Expression

Emotion_Scale	Expression	Mean	SD	CI95 lower	CI95 upper
**Anger**	Negative	3.56	1.27	3.39	3.73
**Anger**	Neutral	1.51	0.85	1.40	1.63
**Anger**	Positive	1.88	0.81	1.78	1.99
**Disgust**	Negative	2.29	1.12	2.14	2.44
**Disgust**	Neutral	1.64	0.83	1.53	1.75
**Disgust**	Positive	1.73	0.75	1.63	1.83
**Fear**	Negative	2.94	1.19	2.78	3.10
**Fear**	Neutral	1.92	1.00	1.78	2.05
**Fear**	Positive	2.17	0.89	2.05	2.28
**Happy**	Negative	2.17	1.11	2.02	2.31
**Happy**	Neutral	1.99	0.99	1.86	2.12
**Happy**	Positive	3.14	1.18	2.98	3.29
**Sad**	Negative	1.79	0.93	1.66	1.91
**Sad**	Neutral	2.31	1.25	2.15	2.48
**Sad**	Positive	1.78	0.77	1.68	1.88
**Surprise**	Negative	2.57	1.09	2.42	2.71
**Surprise**	Neutral	1.71	0.81	1.61	1.82
**Surprise**	Positive	2.41	1.01	2.28	2.55

3 Emotion profile

3.1 Summary statistics

3.2 Graph

```
# Dodge position to separate bars for Femo within each Temo
pd <- position_dodge(width = 0.75)

Profile_graph <- ggplot(desciptives,
                aes(x = Expression, y = mean, fill = Emotion_Scale)) +
  geom_col(position = pd, width = 0.7, color = "grey30") +      # bars
  geom_errorbar(aes(ymin = ci_low, ymax = ci_high),             # your CI
                position = pd, width = 0.15, linewidth = 0.6) +
```

```
  theme_minimal() +
    labs(
       x = "Expression Valence",
       y = "Mean Rating") +
  paletteer::scale_fill_paletteer_d("lisa::FridaKahlo") +
  theme(
    axis.title.x = element_text(margin = margin(t = 20))  # Increase top margin of x-axis titl
  )

plot(Profile_graph)
```

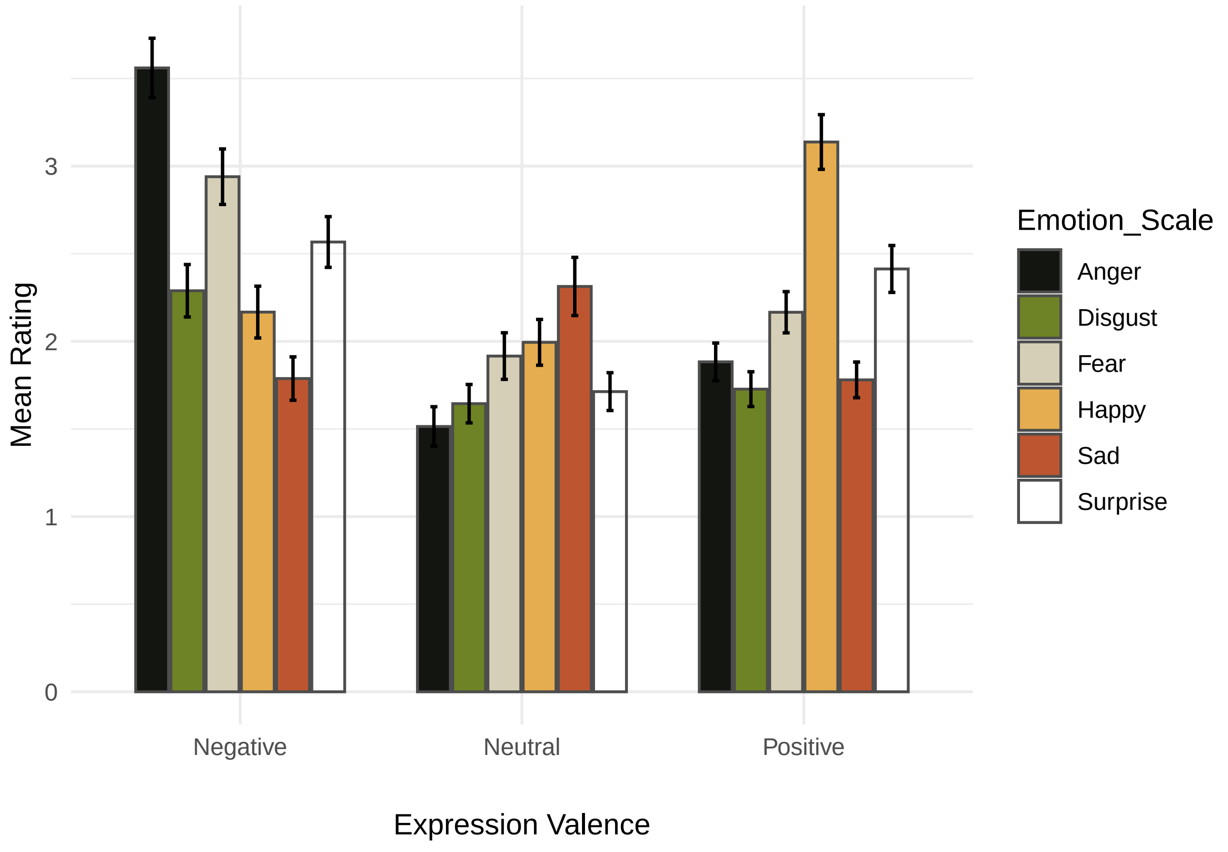

3.3 Analysis and post-hocs

Table 9: ANOVA Results with Partial Eta Squared

	Sum Sq	Mean Sq	NumDF	DenDF	F value	Pr(>F)	Partial ^{2}
Emotion_Scale	777.8558	155.5712	5	18851	69.3306	0	0.0181
Expression	1570.4882	785.2441	2	18851	349.9458	0	0.0358
Emotion_Scale:Expression	3257.7508	325.7751	10	18851	145.1824	0	0.0715

Table 10: Positive Expression Results

	Comparison	Statistics					
	Contrast	Estimate	SE	df	t-ratio	p-value	NA
**31**	Anger - Disgust	Positive	0.156	0.065	18851	2.392	0.159
**32**	Anger - Fear	Positive	-0.283	0.065	18851	-4.350	0.000
**33**	Anger - Happy	Positive	-1.255	0.065	18851	-19.283	0.000
**34**	Anger - Sad	Positive	0.103	0.065	18851	1.580	0.612
**35**	Anger - Surprise	Positive	-0.530	0.065	18851	-8.148	0.000
**36**	Disgust - Fear	Positive	-0.439	0.065	18851	-6.742	0.000
**37**	Disgust - Happy	Positive	-1.410	0.065	18851	-21.676	0.000
**38**	Disgust - Sad	Positive	-0.053	0.065	18851	-0.812	0.965
**39**	Disgust - Surprise	Positive	-0.686	0.065	18851	-10.541	0.000
**40**	Fear - Happy	Positive	-0.972	0.065	18851	-14.934	0.000
**41**	Fear - Sad	Positive	0.386	0.065	18851	5.930	0.000
**42**	Fear - Surprise	Positive	-0.247	0.065	18851	-3.799	0.002
**43**	Happy - Sad	Positive	1.358	0.065	18851	20.864	0.000
**44**	Happy - Surprise	Positive	0.725	0.065	18851	11.135	0.000
**45**	Sad - Surprise	Positive	-0.633	0.065	18851	-9.729	0.000

Note:

Positive expression pairwise comparison results

Table 11: Neutral Expression Results

	Comparison	Statistics					
	Contrast	Estimate	SE	df	t-ratio	p-value	NA
**16**	Anger - Disgust	Neutral	-0.130	0.065	18851	-2.001	0.342
**17**	Anger - Fear	Neutral	-0.402	0.065	18851	-6.176	0.000
**18**	Anger - Happy	Neutral	-0.480	0.065	18851	-7.380	0.000
**19**	Anger - Sad	Neutral	-0.799	0.065	18851	-12.280	0.000
**20**	Anger - Surprise	Neutral	-0.199	0.065	18851	-3.059	0.027
**21**	Disgust - Fear	Neutral	-0.272	0.065	18851	-4.176	0.000
**22**	Disgust - Happy	Neutral	-0.350	0.065	18851	-5.379	0.000
**23**	Disgust - Sad	Neutral	-0.669	0.065	18851	-10.280	0.000
**24**	Disgust - Surprise	Neutral	-0.069	0.065	18851	-1.058	0.898
**25**	Fear - Happy	Neutral	-0.078	0.065	18851	-1.203	0.835
**26**	Fear - Sad	Neutral	-0.397	0.065	18851	-6.104	0.000
**27**	Fear - Surprise	Neutral	0.203	0.065	18851	3.117	0.023
**28**	Happy - Sad	Neutral	-0.319	0.065	18851	-4.901	0.000
**29**	Happy - Surprise	Neutral	0.281	0.065	18851	4.321	0.000
**30**	Sad - Surprise	Neutral	0.600	0.065	18851	9.221	0.000

Note:
Neutral expression pairwise comparison results

Table 12: Negative Expression Results

	Comparison	Statistics				
Contrast	Estimate	SE	df	t-ratio	p-value	NA
**Anger - Disgust**	Negative	1.272	0.065	18851	19.544	0.000
**Anger - Fear**	Negative	0.621	0.065	18851	9.540	0.000
**Anger - Happy**	Negative	1.393	0.065	18851	21.415	0.000
**Anger - Sad**	Negative	1.773	0.065	18851	27.243	0.000
**Anger - Surprise**	Negative	0.993	0.065	18851	15.267	0.000
**Disgust - Fear**	Negative	-0.651	0.065	18851	-10.004	0.000
**Disgust - Happy**	Negative	0.122	0.065	18851	1.870	0.421
**Disgust - Sad**	Negative	0.501	0.065	18851	7.699	0.000
**Disgust - Surprise**	Negative	-0.278	0.065	18851	-4.277	0.000
**Fear - Happy**	Negative	0.773	0.065	18851	11.874	0.000
**Fear - Sad**	Negative	1.152	0.065	18851	17.703	0.000
**Fear - Surprise**	Negative	0.373	0.065	18851	5.727	0.000
**Happy - Sad**	Negative	0.379	0.065	18851	5.828	0.000
**Happy - Surprise**	Negative	-0.400	0.065	18851	-6.147	0.000
**Sad - Surprise**	Negative	-0.779	0.065	18851	-11.976	0.000

Note:
Negative expression pairwise comparison results

4 Participant expression (action units)

.

Table 13: Descriptive statistics by AU and Expression

AU	Expression	Mean	SD	CI95 lower	CI95 upper
**AU04z**	Negative	0.07	0.30	0.03	0.11
**AU06z**	Negative	0.00	0.28	-0.04	0.04
**AU12z**	Negative	0.00	0.27	-0.04	0.04
**AU04z**	Neutral	-0.02	0.31	-0.07	0.02
**AU06z**	Neutral	-0.03	0.32	-0.07	0.02
**AU12z**	Neutral	0.00	0.32	-0.05	0.04
**AU04z**	Positive	-0.02	0.29	-0.06	0.02
**AU06z**	Positive	0.13	0.29	0.09	0.17
**AU12z**	Positive	0.09	0.29	0.05	0.13

4.1 Graph

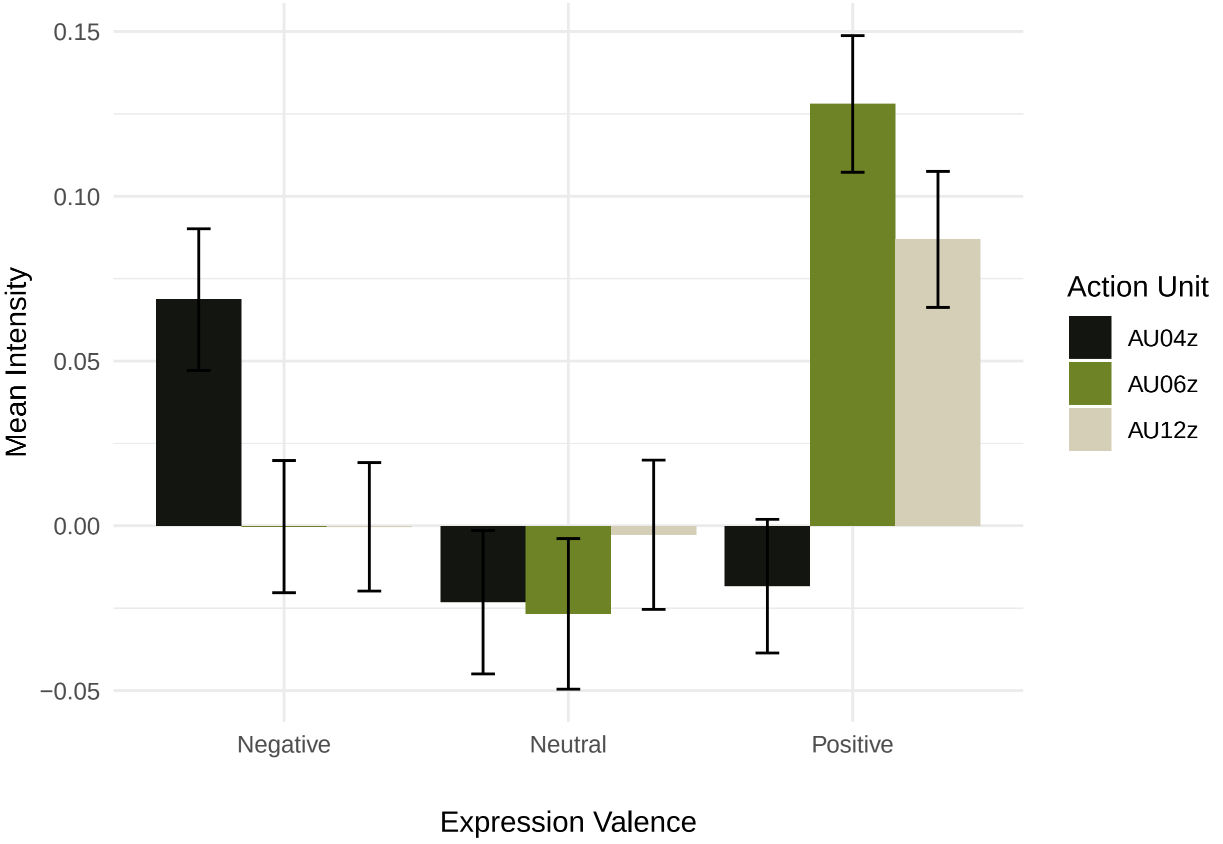

4.2 Analysis

Table 14: ANOVA Results with Partial Eta Squared

	Df	Sum Sq	Mean Sq	F value	Pr(>F)	Partial ^{2}
AU	2	0.9749	0.4875	1.0005	0.3677	0.0003
Expression	2	7.7456	3.8728	7.9487	0.0004	0.0021
AU:Expression	4	10.1021	2.5255	5.1835	0.0004	0.0027

4.3 Post hoc Helmert contrasts

Table 15: Helmert Contrast with Effect Size and Confidence Intervals

	Contrast Estimate					Effect Size	95% CI	
Emotion	Estimate	SE	df	t-ratio	p-value	Partial ^{2}	CI Lower	CI Upper
Negative	-0.122	0.058	7550	-2.085	0.037	0.001	-0.236	-0.007
Neutral	0.020	0.058	7550	0.351	0.725	0.000	-0.093	0.134
Positive	0.236	0.058	7550	4.104	0.000	0.002	0.123	0.349

5 Liking and IOS

5.1 Graph

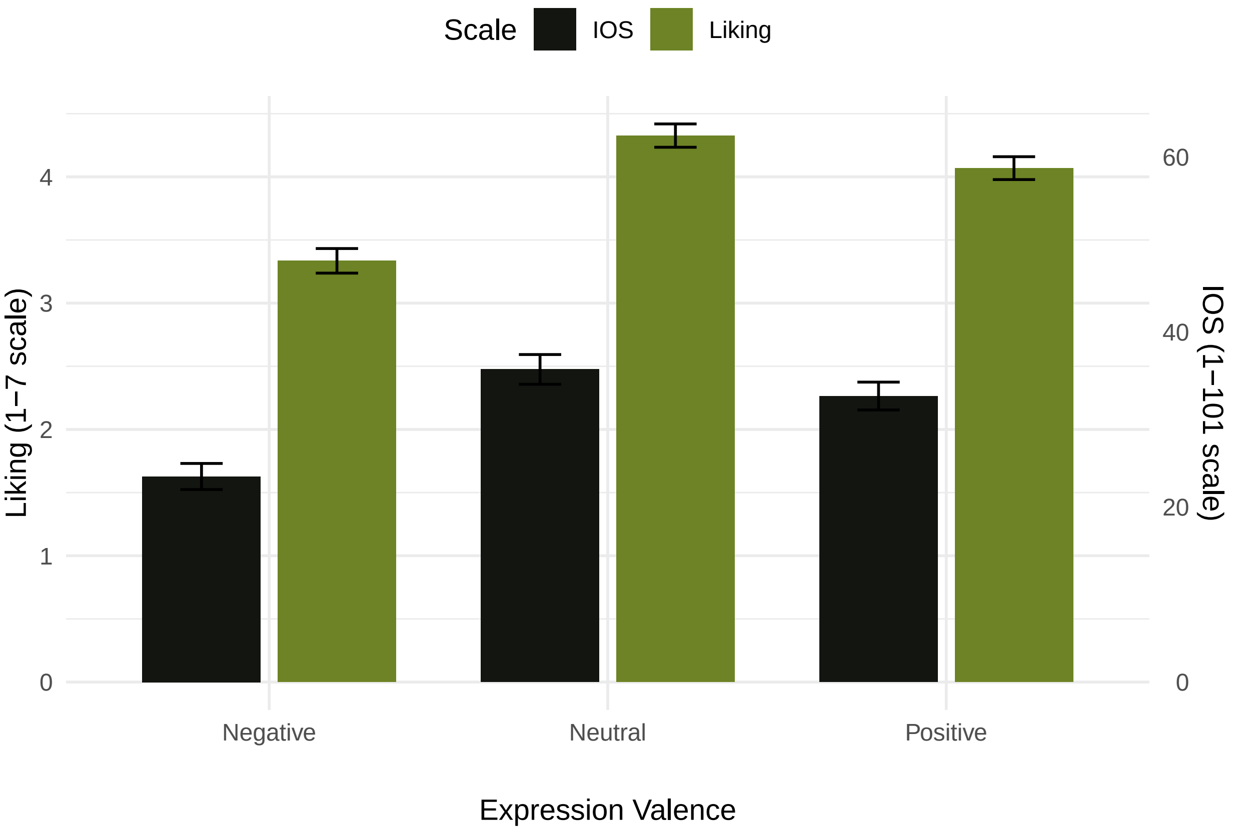

5.2 Summary statistics

Table 16: Descriptive statistics by Scale and Expression

Scale	Expression	Mean	SD	CI95 lower	CI95 upper
**IOS**	Negative	23.48	21.66	20.57	26.40
**IOS**	Neutral	35.71	24.73	32.38	39.04
**IOS**	Positive	32.67	23.18	29.55	35.79
**Liking**	Negative	3.33	1.42	3.14	3.53
**Liking**	Neutral	4.33	1.35	4.15	4.51
**Liking**	Positive	4.07	1.32	3.89	4.25

5.3 Analyses

5.3.1 Liking

Table 17: ANOVA Results with Partial Eta Squared

	Sum Sq	Mean Sq	NumDF	DenDF	F value	Pr(>F)	Partial ^{2}
Expression	362.1643	181.0821	2	1972.221	108.8489	0	0.0994

Table 18: Pairwise Comparisons

Contrast	Estimate	SE	df	t-ratio	p-value
Negative - Neutral	-0.973	0.069	1971.855	-14.096	0.000
Negative - Positive	-0.747	0.069	1971.291	-10.871	0.000
Neutral - Positive	0.226	0.069	1972.410	3.292	0.003

5.3.2 Perceived closeness (IOS)

Table 19: ANOVA Results with Partial Eta Squared

	Sum Sq	Mean Sq	NumDF	DenDF	F value	Pr(>F)	Partial ^{2}
Expression	57098.6	28549.3	2	1970.536	78.0692	0	0.0734

Table 20: Pairwise Comparisons

Contrast	Estimate	SE	df	t-ratio	p-value
Negative - Neutral	-12.033	1.023	1970.837	-11.761	0.000
Negative - Positive	-9.754	1.019	1970.265	-9.575	0.000
Neutral - Positive	2.279	1.017	1971.436	2.241	0.065

6 Predicting the Positive Pattern Score from dimensional emotion ratings

6.1 Summary statistics Positive Pattern Score

6.2 Graph

Table 21: Descriptive statistics by Expression

Expression	Mean	SD	CI95 lower	CI95 upper
**Negative**	-0.06	0.40	-0.11	-0.01
**Neutral**	0.00	0.44	-0.06	0.06
**Positive**	0.13	0.42	0.07	0.18

Table 23: Estimated Marginal Trends for Positive

Trend	Estimate	SE	df	Lower CI	Upper CI	t-value	p-value
overall	0.092	0.011	2166	0.07	0.114	8.316	0

Table 22: ANOVA Results with Partial Eta Squared

	Df	Sum Sq	Mean Sq	F value	Pr(>F)	Partial ^{2}
Positive	1	66.25	66.25	69.1566	0	0.0309

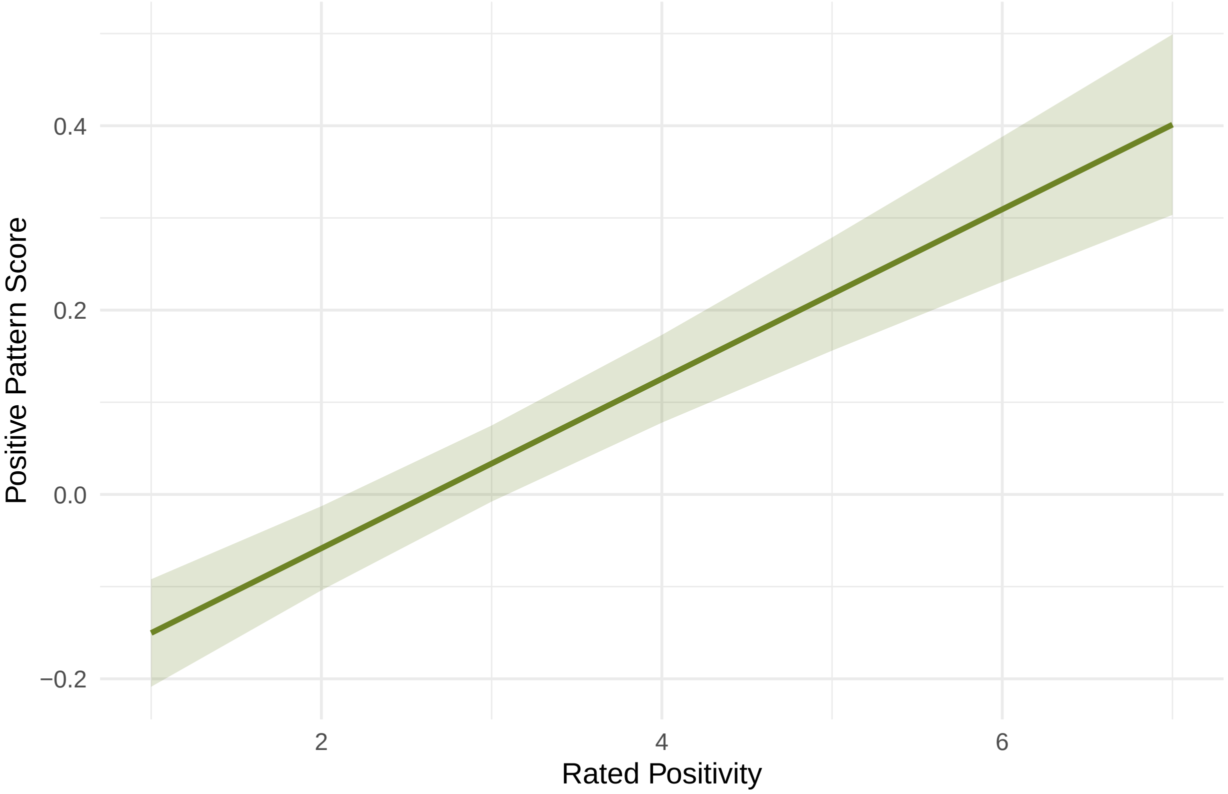

Table 24: Model results

Term	Estimate	SE	t-value	p-value	Partial Eta^2
(Intercept)	0.0003806	0.0221868	0.0171556	0.986	0.000
PositiveZ	0.1303399	0.0233537	5.5811249	< 0.001	0.014
IOSZ	0.0619435	0.0221461	2.7970323	0.005	0.004
PositiveZ:IOSZ	0.0464071	0.0189062	2.4545945	0.014	0.003

7 Using IOS as second predictor

7.1 Analysis

```
##
## Model Summary:  R² = 0.038, Adj. R² = 0.037, F(3, 2164) = 28.52, p = < 0.001, eta² = 0.038
```

7.2 Graph model

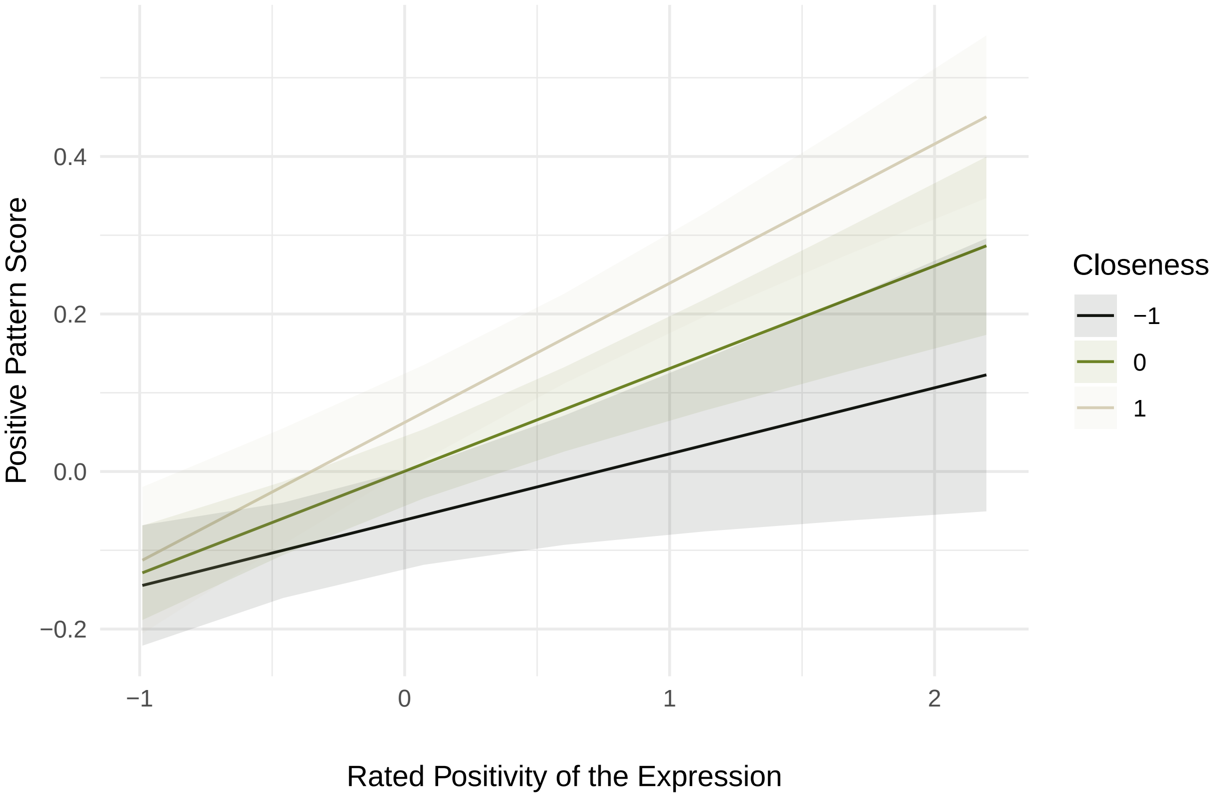

7.3 Post-hoc analysis

Table 25: Estimated Marginal Trends of Positivity at Different Levels of Closeness

	Closeness	Trend Statistics			
IOSZ	PositiveZ.trend	SE	df	lower.CL	upper.CL
**-1**	0.084	0.034	2164	0.017	0.150
**0**	0.130	0.023	2164	0.085	0.176
**1**	0.177	0.026	2164	0.126	0.227

Note:
IOSZ represents standardized levels of closeness: -1 (low), 0 (average), 1 (high)

Table 26: Pairwise Comparisons of Positivity Slopes at Different Levels of Closeness

	Slope Comparison	Statistics				Inference	
contrast	estimate	SE	df	lower.CL	upper.CL	t.ratio	p.value
**(IOSZ-1) - IOSZ0**	-0.046	0.019	2164	-0.091	-0.002	-2.455	0.038
**(IOSZ-1) - IOSZ1**	-0.093	0.038	2164	-0.181	-0.004	-2.455	0.038
**IOSZ0 - IOSZ1**	-0.046	0.019	2164	-0.091	-0.002	-2.455	0.038

Note:
Tukey adjustment applied for multiple comparisons.

8 Powercurve

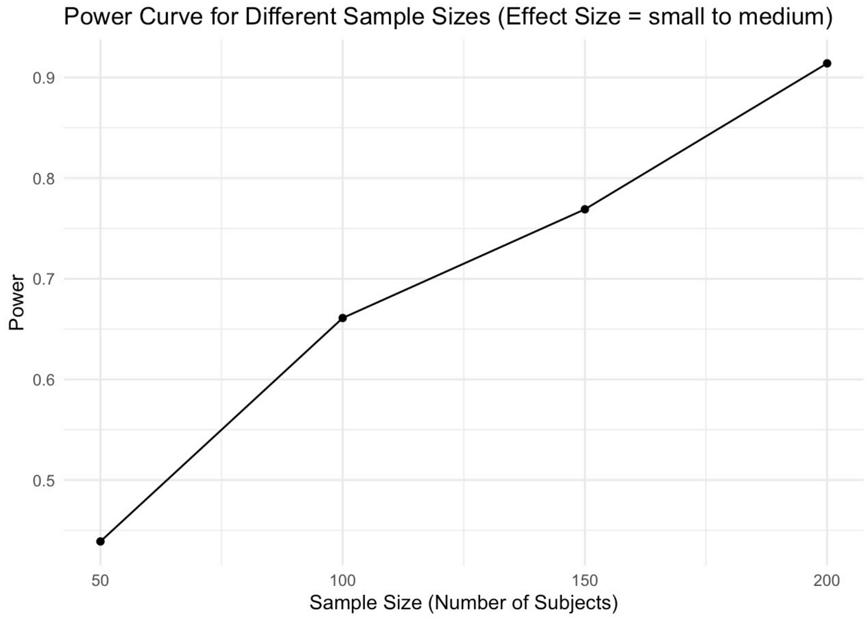

Figure 1: Power curve for a small to medium effect
